# A Prospective Randomized, Controlled, Double-Blind Trial of the Efficacy Using Centella Cream for Scar Improvement

**DOI:** 10.1155/2018/9525624

**Published:** 2018-09-17

**Authors:** Kamonwan Jenwitheesuk, Porntip Rojsanga, Bowornsilp Chowchuen, Palakorn Surakunprapha

**Affiliations:** Division of Plastic and Reconstructive Surgery, Department of Surgery, Faculty of Medicine, Khon Kaen University, Khon Kaen 40000, Thailand

## Abstract

**Objective:**

This study was performed to evaluate the efficacy of* Centella asiatica* extract in cream, a preparation for the prevention of scar development of the split-thickness skin graft (STSG) donor site.

**Methods:**

A prospective randomized, double-blind control study was performed to evaluate the efficacy of Centella cream in 30 patients who underwent a STSG operation. Both Centella cream and placebo were applied equally to the donor site at least 2 weeks after epithelialization was completed. A scar assessment using the Vancouver Scar Scale (VSS) was taken at 4, 8, and 12 weeks.

**Results:**

Of the original 30 patients, 23 patients completed evaluation. There were significant differences in pigmentation parameter of VSS and comparative total VSS scores between 4 and 12 weeks in Centella cream group.

**Conclusion:**

The effect of Centella cream on scar development of a STSG operation may be attainable in terms of better pigmentation. By means of objective measurements and longer follow-up times, Centella cream may prove to be an alternative product for hypertrophic scar amelioration.

## 1. Introduction

Wound healing is a process that takes place with almost all medical treatments. The history of the wound care dates back to the days when natural substances, such as honey and other various remedies, were the norm for treatment since 2600-2200 BCE [1]. Later studies found that natural substances contained in herbs and plants have many positive properties that assist in and enhance the wound healing process, substances such as antioxidants, anti-inflammatories, and antibacterial agents [2]. Many species of plants support the theory that herbs have served a major role in assisting the process of recovery and healing. Studies from different parts of the world have produced concrete findings that support such theories in the era of modern medicine. Evidence stems from research regarding plants and herb usage for treating wounds in different geographical locations, countries such as Africa [3, 4], India [5], China [6] Thai [7], and even the United States, Canada, or Europe [8].

When wounds occur, the chances of a scar forming are greatly increased; scars usually have negative impact on patient. There are many methodologies such as occlusive dressings, compression therapy, intralesional corticosteroid injections, radiation therapy, laser therapy, interferon therapy, and topical silicone gel application, and herbal extracts have been prescribed as the norm. The high cost of these treatments has been the greatest obstacle for patients to continue with treatment, especially in developing countries. “Ugly scars” is a term referring to scar contracture, stretching or hypertrophic scarring resulting from abnormal response by fibroblasts during the proliferative stage and imbalance between collagen synthesis and degradation during the remodeling stage [9]. Nowadays, herbs have an important role in restoring the healing wound process and ameliorating scar. Current studies on* C*.* asiatica *have been conducted to improve the outcome of the wound healing process in small wound types and hypertrophic scar as well as burns, psoriasis, and scleroderma [10–14].

*Centella asiatica*, also commonly known as Gotu kola, is a small plant that depends on the soil with water trapped, as a herbaceous that originates from Asia. It is an annual plant of the family Apiaceae. This plant has been used in folk medicine as well as in western medicine [15]. This study was performed to evaluate the efficacy of* Centella asiatica* extract in a cream preparation for the prevention of scar development on the STSG donor site.

## 2. Methods

### 2.1. Design Overview

All patients who participated consented to join the study on a voluntary basis as the donor site of STSG. Subjects were divided randomly into 2 groups, with various cream applied to each part of the donor's scar site. Cream A (7% w/w* Centella asiatica* extract in cream preparation) or Cream B (placebo) was randomly applied on the subjects. The gels were applied for a total treatment period of 12 weeks. The assessment was conducted by one experienced nurse who was blinded to the subject grouping and was trained to administer all the assessments in standardized manner.

### 2.2. Setting and Participants

From January 2014 to February 2015, 36 patients who underwent split-thickness skin graft harvesting were enrolled in this study, but six patients were excluded because they declined to participate at the Outpatient Unit, Department of Surgery, Faculty of Medicine Srinagarind Hospital. All 30 patients in this study were aged over 20 years and met the inclusion criteria. This study was approved by Khon Kaen University Ethics Committees for Human Research.

*Inclusion Criteria*

1. The donor site of the patients who underwent STSG operation completed more than 14 days of epithelialization.
2. All of the participants were 20 years or older.

*Exclusion Criteria*

1. Patients with critical illnesses such as those with systemic infection or hemodynamic instability.
2. Patients with a major acute or chronic medical illness that could have impact on the wound healing process.
3. Patients who were pregnant.
4. Patients who could not read the Thai language.
5. Patients who declined to participate.

### 2.3. Sample Size

30 voluntary patients were included in this study. The donor site of skin graft in each subject was divided into 2 parts, with different treatments randomly applied to each part. For sample analysis, with 23 subjects per method, the statistical power to remark a mean difference of 1 unit on the rating scale, assuming an SD of 1.71, was 80%. A 2-tailed test with type 1 error rate 5% was supposed. In statistical method, 1.00 corresponded to Mu (M) and 0.02 to the Beta (*β*). Concerning defense against the withdrawal of the patients, it was necessary to raise the number of patients of each group to be 30.

### 2.4. Randomization and Interventions

**Figure 1:**
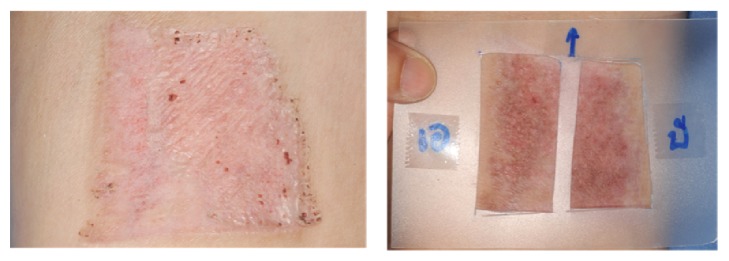
Two-hole template using which patients have to apply the Centella and placebo cream in the frame.

**Figure 2:**
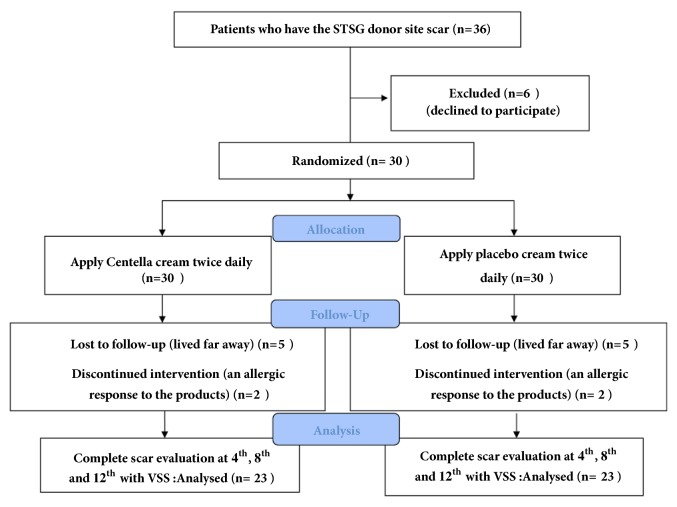
Diagram of study protocol that was initiated.

The* Centella asiatica* was prepared by being extracted with 70% alcohol in cream preparation (Chao Phya Abhaibhubejhr Hospital, Prachin Buri, Thailand). It was formulated from 7% w/w Centella extract, 100 gram, combined with Centella extract 7 g., cetyl alcohol 15 g., stearyl alcohol 12 g., mineral oil 5 g., cetomacrogol-1,000 3 g., propylene glycol 1 g., paraben concentrate 1.5g, and water refill for total of 100 g. for the whole combination. The Centella extract comprised asiaticoside 5.12% and madecassoside 5.1%. The placebo cream was similar in color and consistency to the* Centella asiatica* extract cream and was packed in the same sealed packages. For the composition, it was the same as* Centella asiatica* extract cream except 7 g. of paraben concentrate. Centella and placebo cream were marked A or B. The patients were advised to clean their hands before applying each cream and to use the template (Figure 1). After placing the template on the wound, the patient had to gently apply approximately 1 gram of each cream and wait until they were completely absorbed. This application was done twice daily. Each patient was scheduled for follow-up at 4, 8, and 12 weeks (Figure 2).

### 2.5. Outcomes and Measurements

Patient demographic data were recorded. The scars were examined and rated using the Vancouver Scar Scale (Table 1) to determine pigmentation, vascularity, pliability, and height. The digital photos of each scar were also recorded by using a digital camera each time the assessment was performed, and the photos taken were standardized with fixed distance and lighting. A digital camera was used to ensure that the images taken were clear and comparable.

The Vancouver Scar Scale measured vascularity, pliability, and height, each on a 3- to 6-point ordinal scale; pigmentation was measured on a 3-point categorical scale. The results were recorded and patients identified as to which of the 2 groups they belonged after the protocol was finished. If the treatment decreased in the score of each parameter, the result concluded with improvements. If the score was the same or higher, no response was reported.

### 2.6. Statistical Analysis

Greenhouse-Geisser correction (p value) and one-way repeated measures ANOVA were used to assess two more groups who were participants in the same group. All statistical analyses were operated using SPSS 16.0 software (SPSS, Chicago, IL.). A p value equal to or below 0.05 was regarded as statistically significant

## 3. Results

**Figure 3:**
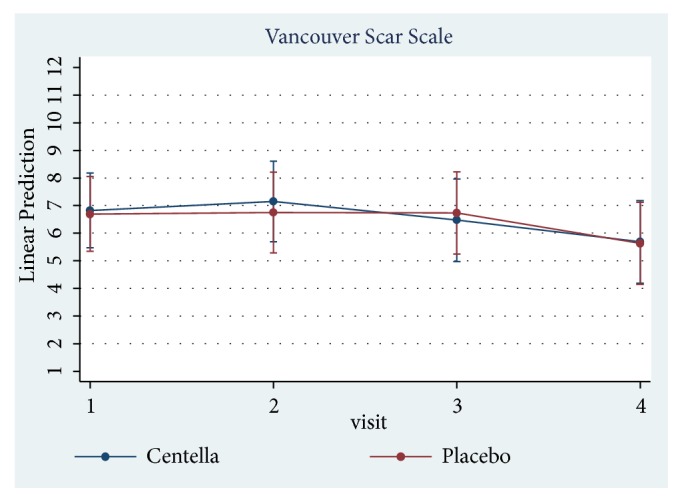
Comparison of Vancouver scar scores within groups after 12 weeks.

**Figure 4:**
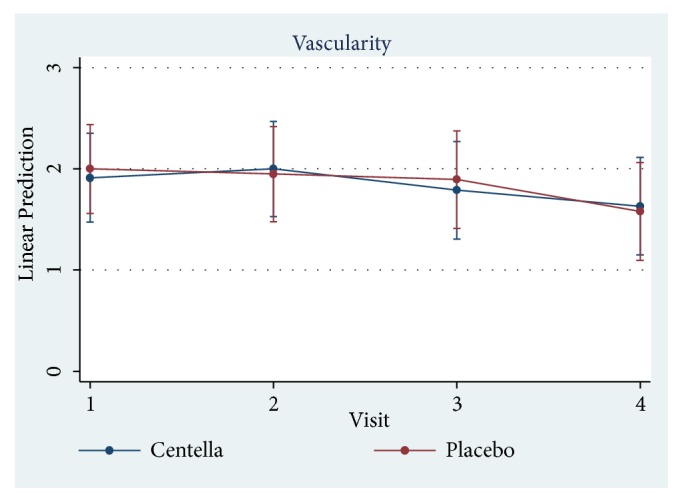
Comparison of vascularity scores within groups after 12 weeks.

**Figure 5:**
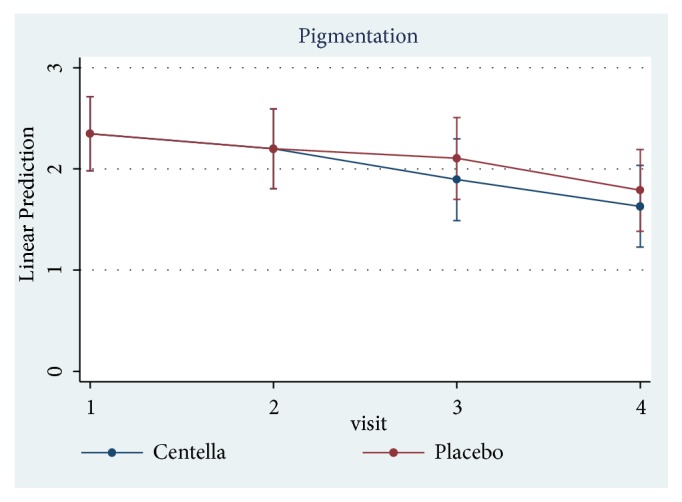
Comparison of pigmentation scores within groups after 12 weeks.

**Figure 6:**
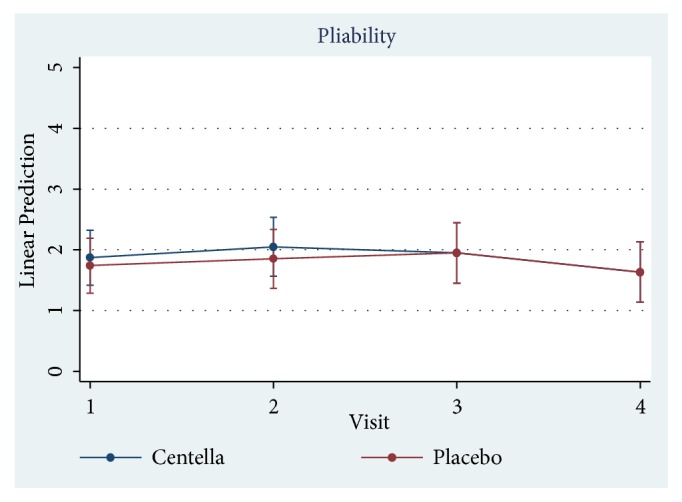
Comparison of pliability scores within groups after 12 weeks.

**Figure 7:**
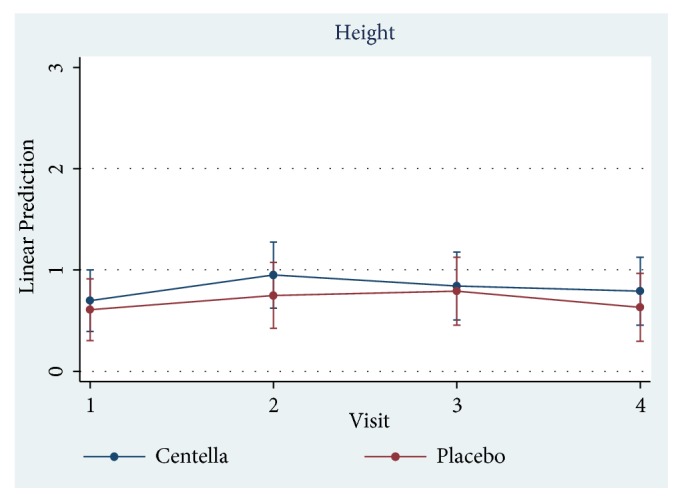
Comparison of height scores within groups after 12 weeks.

Only 23 of 30 patients completed the study protocol. 2 patients were excluded due to rash at the scar, which may have been caused by an allergic response to the products. 5 patients were lost to follow-up because they lived far away. Of 23 patients, there were 13 males and 10 females. The average age was 54 years old (range: 20-65 years old). As shown in Table 2 and Figures 3–7, differences within groups are as follows: For the Centella cream group, there were differences from baseline including the pigmentation score at 8 and 12 weeks and between 4 and 12 weeks (-0.443, p value 0.019; -0.707, p value 0.001; -0.557, p value 0.001) and the overall Vancouver Scar Scale scores between 4 and 12 weeks (-1.279, p value 0.041). However, for height, it was worse at 4 weeks (0.300, p value 0.043). For the placebo group, there were 2 differences from the baseline including the pigmentation score between 4 and 12 weeks (-0.399, p value 0.020) and after 12 weeks (-0.549, p value 0.002), while the pliability and the height scores of both groups were compared before and after treatment and still were not different.

## 4. Discussion

The split-thickness skin graft donor site is a superficial partially thickness wound in which losing the epidermis and part of the dermis exists. Epithelialization is the natural act of healing dermal tissue resulting in minimal or no scarring [16]. In most cases, scars occur if the depth reaches the dermis layer; the exposed area of the scar can be more problematic. Treatment for scars can be difficult, and in some cases the skin cannot return to its normal condition causing additional suffering to the patient. Some patients complained about discoloration, both hyperpigmentation and hypopigmentation. When hypertrophic scar or keloid developed, it may induce itching, pain, and uninviting scar damage, sometimes in the form of scar contracture which may cause organ dysfunction. In some cases, patients were unhappy and expressed a great deal of regret, anger, rejection, and isolation despair [17]. The knowledge of scar protection/prevention and causes of the scar is an important factor and helps reduce its severity. However, some Asians displayed a condition referred to as “Fitzpatrick Skin” (type III or type IV): commonly known as hypervascular, hyperpigmented, hypopigmented, or hypertrophic scars when other related characteristics are the anatomic region, patient's skin type or genetic factors, nature of injury, skin tension, and prolonged inflammatory process [18]. Numerous attempts have been made to introduce natural substances to reduce scars such as onion extract [19, 20], resveratrol in grape's skin [21], curcumin [22], and* Centella* [23].

Scar protection applications can be made by starting from coagulation, inflammation, and proliferation phase, which tried to make each phase without any complications and was time consuming. In the remodeling phase, collagen was rearranged and was broken by enzyme matrix metalloproteinase (MMPs), which is controlled by tissue inhibitors of MMP (TIMPs) [24]. The balance between regeneration and degradation of collagen was initiated during the time periods of 6 months to 1 year. The scar was entirely abnormal in appearance, the architectural arrangement of collagen, and ECM. The strategy for minimized scar needed many factors like efficiently control inflammation [25] and low levels of cytokines such as TGF *β*I, TGF *β*II, and platelet-derived growth factor [26, 27] but high levels of TGF *β* III [26–28]. TGF *β* I and TGF *β* II played a role in activating proliferation of fibroblasts, whereas TGF *β* III was antagonist and that is why it could prevent scar formation [29].

**Figure 8:**
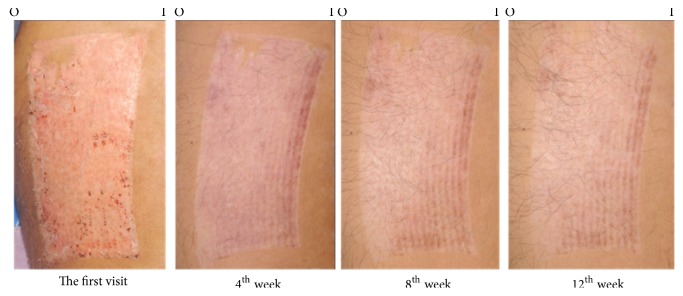
A 58-year-old male patient with a split-thickness skin graft harvested from right thigh. Centella cream was applied at the outer part with placebo at the inner part of thigh (I: inner part, O: outer part).

**Figure 9:**
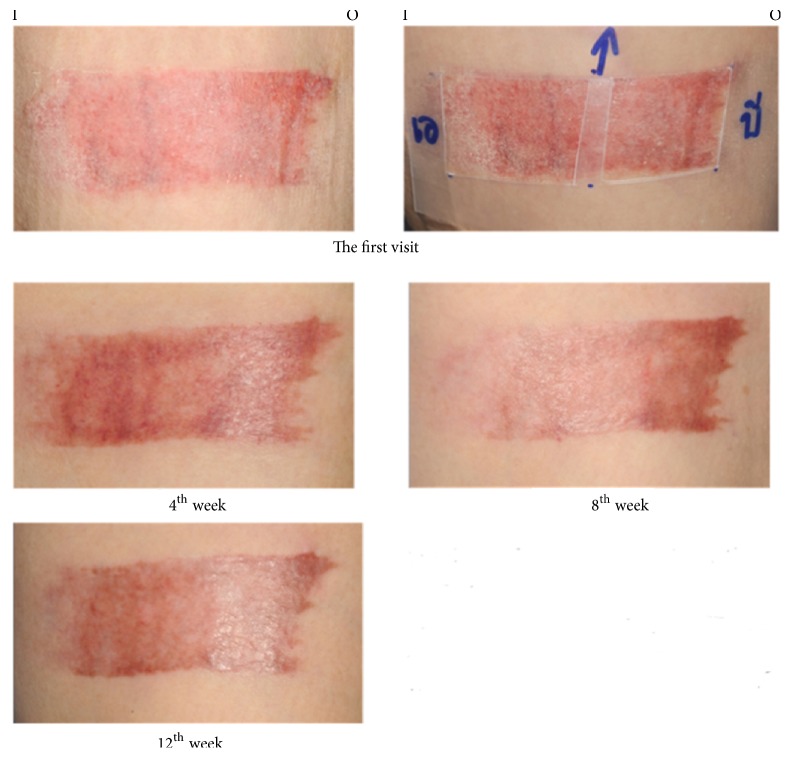
A 66-year-old female patient with a split-thickness skin graft harvested from left thigh. Centella cream was applied at the inner part with placebo at the outer part of thigh (I: inner part, O: outer part).

Prior studies have shown the effectiveness of* C. asiatica *extract in promotion of wound healing and prevention of hypertrophic scars [19, 30, 31]. The active compounds of* C. asiatica* responsible for those activities are pentacyclic triterpenes, including asiaticoside, and madecassoside. In vitro study demonstrated that asiaticoside decreased fibroblast proliferation in a dose-related manner and reduced the expression of both TGF- *β* I and TGF- *β* II at the transcriptional and translational level [32]. Asiaticoside also slows down scar formation possibility by increasing the activity process of Smad7 which is a negative regulator of TGF-*β* signaling [33]. The other active composition, madecassoside, is related to inhibiting the migration of fibroblasts from keloids [34]. Both active chemical substances promote* C. asiatica* to induce fibroblast proliferation and collagen synthesis. It involves the improvement of the tensile strength of newly formed skin and stimulation of maturation of the scar by the production of type I collagen. Inversely, the expression of TGF-*β*I in hypertrophic scars and keloid is reduced [32, 34, 35]. Both asiaticoside and madecassoside affected the healing process mechanism in which inflammatory, proliferation, and remodeling phases initiate the improvement of wound healing and scar prevention [10]. In the case of animal research they showed effective result of diminished hypertrophic scar [30]. Numerous data confirmed that* C*.* asiatica *extract in a combination form with other herbs could prevent abnormal scars from median sternotomy or split-thickness skin graft donor sites; however, no exact demonstration revealed that one was stronger for preventing scar [19, 31]. The aim of this study was to evaluate the efficacy of Centella cream with only one chemical substance, not combined with other herbal substances, on the prevention of scar development. Our study revealed that Centella cream improved scar outcomes against placebo. The Centella cream significantly improved the overall Vancouver scores between 4 and 12 weeks and pigmentation from the baseline since 8th week, in comparison with the improved pigmentation from the baseline since the 12th week in the placebo group. This evidence concluded that the nature of scar development of the donor site of the split- thickness skin grafts got better over time, ameliorating by Centella effect. Figures 8 and 9 demonstrated the better outcome in skin pigmentation after applying the Centella cream. In this study, an allergic dermatitis was observed in 2 patients. Gomes reported in the reported case that the patient had contact dermatitis due to* Centella asiatica* extract [36]. Since the allergic reaction occurred with both Centella cream and placebo, the ingredients of the placebo may be responsible for the allergic dermatitis. Previous reports showed that contact dermatitis developed when paraben-containing products were applied [37].

The efficacy of* C. asiatica* formulation may be affected by the release of active compounds from the formulation. The animal (rat) model demonstrated the promoted cellular proliferation and collagen synthesis effects of the aqueous extract* C. asiatica.* The gel formulation provided significantly better healing outcome than the ointment and cream formulations [38].

The VSS is more subjective measurement. Studies with longer follow-ups may be required to confirm the benefits of this product. In the future, the dosage and the manner evaluation of efficacy of* Centella asiatica* need standardized experimentation in the healing process and scar prevention.

## 5. Conclusion

The effect of Centella cream on scar development of the donor site of the split- thickness skin grafts may be attainable in terms of better pigmentation. By means of objective measurements and longer follow-up times, Centella cream may prove to be an alternative product for hypertrophic scar amelioration.

## Figures and Tables

**Table 1:** Vancouver Scar Scale (VSS).

**Score**	**0**	**1**	**2**	**3**	**4**	**5**
Vascularity	Normal	Pink	Red	Purple		
Pigmentation	Normal	Hypopigmentation	Mixed	Hyperpigmentation		
Pliability	Normal	Supple	Yielding	Firm	Ropes	Contractures
Height	Flat	<2 mm	2-5 mm	>5 mm		

**Table 2:** Comparison within groups after 12 weeks of both groups.

**Vancouver Scar Scale**	**Centella cream**	**Contrast**	**95% CI**	**P-value**
After	week 4th	0.400	-0.799 - 1.599	0.510
After	week 8th	-0.089	-1.312 - 1.133	0.885
After	week 12th	-0.879	-2.102 - 0.344	0.157
Between	8&4 weeks	-0.489	-1.712 - 0.733	0.429
Between	12&4 weeks	-1.279	-2.502 - -0.056	0.041^*∗*^
Between	12&8 weeks	-0.789	-2.020 - 0.441	0.206

**Vancouver Scar Scale**	**Placebo**			

After	week 4th	0.150	-1.049 - 1.349	0.805
After	week 8th	0.312	-0.911 - 1.535	0.614
After	week 12th	-0.793	-2.016 - 0.429	0.201
Between	8&4 weeks	0.162	-1.061 - 1.385	0.794
Between	12&4 weeks	-0.943	-2.166 - 0.279	0.129
Between	12&8 weeks	-1.105	-2.336 - 0.125	0.078

**Vascularity**	**Centella cream**			

After	week 4th	0.100	-0.426 - 0.626	0.707
After	week 8th	-0.055	-0.591 - 0.481	0.838
After	week 12th	-0.213	-0.749 - 0.323	0.432
Between	8&4 weeks	-0.155	-0.691 - 0.381	0.567
Between	12&4 weeks	-0.313	-0.849 - 0.223	0.249
Between	12&8 weeks	-0.158	-0.697 - 0.381	0.563

**Vascularity**	**Placebo**			

After	week 4th	-0.050	-0.576 - 0.476	0.851
After	week 8th	-0.051	-0.587 - 0.485	0.850
After	week 12th	-0.367	-0.903 - 0.169	0.177
Between	8&4 weeks	-0.001	-0.537 - 0.535	0.996
Between	12&4 weeks	-0.317	-0.853 - 0.219	0.244
Between	12&8 weeks	-0.316	-0.855 - 0.224	0.248

**Pigmentation**	**Centella cream**			

After	week 4th	-0.150	-0.478 - 0.178	0.366
After	week 8th	-0.443	-0.777 - -0.109	0.010^*∗*^
After	week 12th	-0.707	-1.041 - -0.373	0.001^*∗*^
Between	8&4 weeks	-0.293	-0.627 - 0.041	0.084
Between	12&4 weeks	-0.557	-0.891 - -0.223	0.001^*∗*^
Between	12&8 weeks	-0.263	-0.599 - 0.073	0.124

**Pigmentation**	**Placebo**			

After	week 4th	-0.150	-0.478 - 0.178	0.366
After	week 8th	-0.233	-0.567 - 0.101	0.170
After	week 12th	-0.549	-0.883 - -0.215	0.002^*∗*^
Between	8&4 weeks	-0.083	-0.417 - 0.251	0.624
Between	12&4 weeks	-0.399	-0.733 - -0.065	0.020^*∗*^
Between	12&8 weeks	-0.316	-0.652 - 0.020	0.065

**Pliability**	**Centella cream**			

After	week 4th	0.200	-0.202 - 0.602	0.326
After	week 8th	0.179	-0.231 - 0.589	0.389
After	week 12th	-0.137	-0.547 - 0.273	0.509
Between	8&4 weeks	-0.021	-0.431 - 0.389	0.919
Between	12&4 weeks	-0.337	-0.747 - 0.073	0.106
Between	12&8 weeks	-0.316	-0.728 - 0.097	0.132

**Pliability**	**Placebo**			

After	week 4th	0.150	-0.252 - 0.552	0.461
After	week 8th	0.312	-0.098 - 0.722	0.134
After	week 12th	-0.004	-0.414 - 0.406	0.985
Between	8&4 weeks	0.162	-0.248 - 0.572	0.435
Between	12&4 weeks	-0.154	-0.564 - 0.256	0.458
Between	12&8 weeks	-0.316	-0.728 - 0.097	0.132

**Height**	**Centella cream**			

After	week 4th	0.300	0.009 - 0.591	0.043^*∗*^
After	week 8th	0.229	-0.067 - 0.525	0.129
After	week 12th	0.176	-0.120 - 0.473	0.241
Between	8&4 weeks	-0.071	-0.367 - 0.225	0.636
Between	12&4 weeks	-0.124	-0.420 - 0.173	0.410
Between	12&8 weeks	-0.053	-0.351 - 0.246	0.727

** Height**	**Placebo**			

After	week 4th	0.200	-0.091 - 0.491	0.176
After	week 8th	0.284	-0.012 - 0.581	0.060
After	week 12th	0.126	-0.170 - 0.423	0.400
Between	8&4 weeks	0.084	-0.212 - 0.381	0.575
Between	12&4 weeks	-0.074	-0.370 - 0.223	0.623
Between	12&8 weeks	-0.158	-0.456 - 0.140	0.296

## Data Availability

The data used to support the findings of this study are available from the corresponding author upon request.
